# Risk factors for operated carpal tunnel syndrome: a multicenter population-based case-control study

**DOI:** 10.1186/1471-2458-9-343

**Published:** 2009-09-16

**Authors:** Stefano Mattioli, Alberto Baldasseroni, Massimo Bovenzi, Stefania Curti, Robin MT Cooke, Giuseppe Campo, Pietro G Barbieri, Rinaldo Ghersi, Marco Broccoli, Maria Pia Cancellieri, Anna Maria Colao, Marco dell'Omo, Pirous Fateh-Moghadam, Flavia Franceschini, Serenella Fucksia, Paolo Galli, Fabriziomaria Gobba, Roberto Lucchini, Anna Mandes, Teresa Marras, Carla Sgarrella, Stefano Borghesi, Mauro Fierro, Francesca Zanardi, Gianpiero Mancini, Francesco S Violante

**Affiliations:** 1 Occupational Medicine Unit, Dipartimento di Medicina Interna, dell'Invecchiamento e Malattie Nefrologiche, University of Bologna, Bologna, Italy; 2 Tuscany Regional Centre for Occupational Injuries and Diseases (CeRIMP), Florence, Italy; 3 Institute of Occupational Medicine, University of Trieste, Trieste, Italy; 4 Dipartimento Processi Organizzativi, National Institute of Occupational Safety and Prevention (ISPESL), Rome, Italy; 5 Occupational Health Service, ASL di Brescia, Brescia, Italy; 6 Occupational Health Service, ASL di Modena, Modena, Italy; 7 Occupational Health Service, ASL di Ravenna, Ravenna, Italy; 8 Occupational Health Service, ASUR Marche - Zona 2, Urbino, Italy; 9 Occupational Health Service, ASUR Marche - Zona 6, Fabriano, Italy; 10 Institute of Occupational Medicine and Toxicology, University of Perugia, Perugia, Italy; 11 Epidemiology Unit, Azienda Provinciale per i Servizi Sanitari, Provincia Autonoma di Trento, Trento, Italy; 12 Occupational Health Service, ASL di Bologna, Bologna, Italy; 13 Occupational Health Service, ASL di Imola, Imola, Italy; 14 Department of Public Health Sciences, University of Modena and Reggio Emilia, Modena, Italy; 15 Occupational Medicine Institute, University of Brescia, Brescia, Italy; 16 Occupational Health Service, ASL di Sassari, Sassari, Italy; 17 Occupational Health Service, ASL di Firenze, Florence, Italy

## Abstract

**Background:**

Carpal tunnel syndrome (CTS) is a socially and economically relevant disease caused by compression or entrapment of the median nerve within the carpal tunnel. This population-based case-control study aims to investigate occupational/non-occupational risk factors for surgically treated CTS.

**Methods:**

Cases (n = 220) aged 18-65 years were randomly drawn from 13 administrative databases of citizens who were surgically treated with carpal tunnel release during 2001. Controls (n = 356) were randomly sampled from National Health Service registry records and were frequency matched by age-gender-specific CTS hospitalization rates.

**Results:**

At multivariate analysis, risk factors were blue-collar/housewife status, BMI ≥ 30 kg/m^2^, sibling history of CTS and coexistence of trigger finger. Being relatively tall (cut-offs based on tertiles: women ≥165 cm; men ≥175 cm) was associated with lower risk. Blue-collar work was a moderate/strong risk factor in both sexes. Raised risks were apparent for combinations of biomechanical risk factors that included frequent repetitivity and sustained force.

**Conclusion:**

This study strongly underlines the relevance of biomechanical exposures in both non-industrial and industrial work as risk factors for surgically treated CTS.

## Background

Carpal tunnel syndrome (CTS) is a socially and economically relevant disease caused by compression or entrapment of the median nerve within the carpal canal [1]. Probable risk factors for CTS include age [2], female gender [3], diabetes [4], hypothyroidism [5], obesity [6], family history of CTS [7], menopause [8] and concurrent pathologies such as rheumatoid arthritis [9]. Other factors that have been studied include low height [10,11], smoking history [5], high parity [8], use of oral contraceptives [12], wrist fractures [9] and household chores [13]. Analysis of data from the population-based Occupational Supplement of the U.S. National Health Interview survey indicated that repetitive bending/twisting of the hands/wrists and use of vibratory tools are important risk factors for CTS [14,15]. Moreover, a recent systematic review that considered many cross-sectional studies and some longitudinal/case-control studies found evidence of increased risk of CTS among workers exposed to regular/very repetitious wrist flexion/extension (especially when accompanied by a forceful grip) or to regular/prolonged use of hand-held vibratory tools [16]. Knowledge of the risks associated with job titles is limited (mainly deriving from cross-sectional studies) [17,18]. Few population-based case-control studies looking at both non-occupational and occupational risk factors for CTS are available [19,20].

We performed a multicenter population-based case-control study on risk for surgically treated CTS designed to assess occupational factors (including job titles) alongside proposed non-occupational risk factors.

## Methods

### Selection of participants

Thirteen centers (local administrative authorities from central-northern Italy and Sardinia) participated in the study: the Provinces (*Province*) of Brescia, Modena, Perugia, Ravenna, Sassari and Trent; the Municipalities (*Comuni*) of Bologna and Florence; the Local Health Authorities (*Unità Sanitarie Locali*) of Fabriano, Imola, Urbino, and 'northern Bologna' and 'southern Bologna' (at the time, covering geographical areas to the north and south of the municipality). Each center identified 20 'cases' and 40 'controls', aged 18-65 yr. Identification of 'cases' was based on randomized sampling from their administrative databases containing discharge records from all local hospitals. In Italy, all public and private hospitals (including day-hospitals) are legally obliged to transmit to local authorities individual discharge records containing codified data for compulsory registration in administrative databases based on the patient's residence (irrespective of hospital location). The sampling frame comprised all residents who during the year 2001 had been admitted to hospital (irrespective of the place of treatment) with CTS as the principal diagnosis according to the International Classification of Diseases (ICD-9 code 3540) and who had been submitted to surgical treatment for carpal tunnel release (Diagnosis Related Group [DRG] code 06). At the time of the study, Italian regulations required that carpal tunnel release operations be conducted only on hospital premises. All patients undergoing this treatment had to be formally admitted to hospital--even if only for a few hours on an outpatient basis. Furthermore, diagnostic confirmation by nerve conduction studies was generally considered a prerequisite for carpal tunnel release. Selection of controls was based on random sampling from the national health service registries (*Anagrafe Assistiti Servizio Sanitario Nazionale*) covering each of the thirteen administrative centers included in the study (after frequency matching by age and gender. Of note, at the time of the study all subjects resident in Italy were automatically entitled to national health service membership. Each center received standardized instructions (from S.M. and A.B.) for frequency matching criteria, based on reported age-sex-specific rates of hospitalization for CTS [21] in conjunction with a database regarding incidence of surgically treated CTS in the general population of seven Italian Regions [22]. In particular, each Epidemiology Unit randomly drew 40 controls (32 women, 8 men) in eight age-sex categories (18-34 yr: 4 women, 0 men; 35-44 yr: 6 women, 2 men; 45-54 yr: 14 women, 2 men; 55-65 yr: 8 women, 4 men). Control subjects who had received surgical treatment for CTS were excluded. For both cases and controls, randomization was independently conducted by local Epidemiology Units. All participants provided informed consent. The study protocol was centrally approved by a Local Ethical Committee (Policlinico S. Orsola-Malpighi, Bologna) and conducted in accordance with the guiding principles of the 2004 version of the Declaration of Helsinki.

### Design of questionnaire

We developed a structured questionnaire designed for assessment of a series of potential occupational and non-occupational risk factors, based on those proposed in the literature. Requested information included date of birth; gender; height (cm); weight (kg); level of education (less than elementary school, elementary school, junior high school certificate, high school diploma, university degree); smoking status ('current' [cigarettes/day and duration in yr], 'ex' [with year of cessation], or 'never'), alcohol consumption (3 drinks/day or more, 1-2 drinks/day, 3-6 drinks/wk, 1-2 drinks/wk, or 'never'); current sporting activities and hobbies (with titles and duration in yr); household chores (h/day); family history of CTS (specifying first degree relatives affected and occupations); medical history (for wrist fractures, diabetes mellitus, amyloidosis, gout, progressive systemic sclerosis, rheumatoid arthritis, systemic lupus erythematosus, thyroid disorders, trigger finger, and chronic renal failure); occupational history (previous and current job titles, together with task descriptions; year of retirement, if appropriate). Regarding biomechanical factors, participants were asked to specify for each (previous/current) job, whether it entailed 1) using hand-held vibratory tools; 2) making sustained forceful hand/wrist movements; 3) very frequent repetitive hand/wrist movements; 4) frequent movements in uncomfortable hand postures; 5) frequent pinching actions; 6) manual work provoking skin compression (ie reddening, calluses, blisters or boils). Additionally, women were asked to specify use of oral contraception ('current' [duration in yr], 'past' [with year of cessation], or 'never'); parity (with delivery years); menopause (with year). 'Cases' were asked to provide information on any previous surgery for CTS (specifying affected side and year); occupation at the time of onset of the CTS symptoms that led to surgery in 2001; subsequent change of job due to CTS [yes/no]; any post-intervention problems in resuming daily/occupational activities. 'Controls' had to specify any history of surgical treatment for CTS (to avoid inclusion of inappropriately identified controls). Before the present study, the entire questionnaire was piloted on a sample of 80 subjects with/without CTS [23].

### Administration of questionnaire

The questionnaire was mailed in 2003 to addresses of all 780 participants (twice, when necessary). When a mailed response was not received, we tried to administer the questionnaire by phone: in each center, these interviews were conducted by a single trained interviewer who transcribed participants' responses to identical questionnaires without eliciting additional information. Participants who were still reluctant to collaborate were asked to respond to a brief questionnaire, which requested date of birth, gender, level of education, current/last job title, and reason for not replying to the main questionnaire. When no interview was feasible, the participant was classified as a non-respondent. For deceased subjects and those too ill to answer, the next of kin filled in the written questionnaire (or provided telephone responses). The questionnaire administration phase of the study was closed in 2004.

### Codification of questionnaire

Job titles were coded (according to the European Union variant of the International Standard Classification of Occupations ISCO 88) [24] by three occupational physicians (A.B., M.F., S.M.) who were blind to case/control status. We chose to consider in the main analysis the prevalent job during the past 2 years or--for cases who changed task due to CTS--at the time of onset of symptoms (since a relatively brief exposure period seems to be sufficient to precipitate onset of CTS symptoms) [25]. The only unemployed participant (a control, who was formerly a blue-collar worker) was included in a miscellaneous blue-collar category comprising less frequent job titles. The only student participant (a control) was included in the white-collar category. Retired workers were categorized as ex-blue-/ex-white-collar based on their last job title. Computer use was inferred from the task descriptions reported in the questionnaire. Job-specific plausibility of each of the self-reported biomechanical risk factors was reviewed by a team of three occupational physicians with expertise in ergonomics (M.B., P.G.B., R.G.), who were blinded to case/control status. In addition to job titles and task descriptions, the team took into account age, gender, job-specific employment duration, and historical context. Self-reported risk factors that were considered implausible were reclassified for analytic purposes as "no exposure"; any implausible absence of exposure was also corrected.

### Statistical analysis

After exclusion of non-eligible subjects, participants who responded to the full written questionnaire entered the main analysis, with the exception of retired workers who were considered in a separate analysis because of the absence of current professional exposure. For the main analysis of non-retired subjects, univariate analysis included the main occupational and non-occupational factors. Body mass index (BMI), family history of CTS, alcohol consumption, smoking status, parity, broad socio-occupational groupings (blue-collar, white-collar, housewives) and job title (prevalent in the last 2 years, or at the time of onset of symptoms) were considered as categorical variables. We grouped women's job titles into 15 categories (for men, meaningful job-title analysis was not feasible due to number limitations). Height and education were considered as binary variables, as were each of the co-existent diseases and biomechanical risk factors. Cut-offs for height of women and men were based on the upper tertiles of controls. The cut-off for level of education was high school certificate, which in Italy is conferred at ~19 years of age. We entered variables that reached p < 0.1 at univariate analysis in unconditional logistic regression models constructed to assess risk associated with: 1) broad socio-occupational categories (blue-collar/housewives vs. white-collar) alongside individual factors; and 2) women's job title categories (after adjusting for individual factors). Level of education was excluded from the models due to its strong association with broad socio-occupational groupings. White-collar workers were taken as the reference category for assessment of risks associated with broad socio-occupational categories and job titles. We estimated OR and 95% confidence interval (95%CI) according to Breslow and Day [26]. For the general model including all non-retired women and men (reported in Table 1), we decided to group housewives together with blue-collar workers, based on the rationale that the biomechanical work performed by full-time housewives is broadly analogous to that experienced in certain non-industrial blue-collar jobs such as domestic cleaners or waiters. Based on age-/gender-related frequency differences in the frequency of CTS [9], we conducted separate multivariate analyses for women and men, and also for different age classes (based on tertiles; feasible only for women). Parity was considered only in analyses restricted to women. Number considerations led us to exclude some variables from certain models. For example, in the analysis of women by age groups, we had to exclude alcohol consumption, family history of CTS, several coexistent pathologies and parity. Whenever appropriate, we additionally adjusted for the frequency-matched variables (age and gender) to minimize residual confounding [27]; we also adjusted for center. For selected factors, we calculated the population attributable risk (PAR) with 95% CI using the method described by Natarajan *et al *[28]. From the ergonomic standpoint, we analyzed the plausible self-reported biomechanical risk factors by constructing two unconditional logistic regression models assessing the risks associated with 1) different numbers and 2) various combinations of the six factors. For both these models, the reference category was absence of any plausible biomechanical risk factor. The combinations of risk factors we examined were based on frequency and ergonomic considerations, such as the relevance of force and repetitivity of hand/wrist movements [29]. We also constructed a model crossing ≥1 plausible self-reported biomechanical factors (as a marker of biomechanical exposure) with blue-collar/housewife and white-collar status (taking white-collar workers without biomechanical factors as the reference category). The separate analysis of retired workers was based on multivariate assessment of blue- vs white-collar (reference category) history, again using an unconditional logistic regression model, after adjusting for individual factors. Of note, the decision to focus only on past occupational history was based on number considerations. Stata 9.0 SE (Stata Corporation, Texas, TX) was used for analysis.

**Table 1:** Risk factors for surgically treated CTS among non-retired subjects

			**Univariate**	Multivariate*
			
	**Cases** **(n = 191)**	**Controls** **(n = 286)**	**OR (95% CI)**	**OR (95% CI)**
Socio-occupational status				
White collar	19	134	1.0	1.0
Blue collar/housewife†	172	152	8.0 (4.5-14.2)	7.1 (4.0-12.7)
BMI (kg/m^2^)				
<25	80	167	1.0	1.0
25-29	69	91	1.6 (1.0-2.4)	1.4 (0.9-2.4)
≥30	41	25	3.4 (1.9-6.1)	3.3 (1.6-6.6)
Height, cm				
<165 (women) or <175 (men)	149	165	1.0	1.0
≥165 (women) or ≥175 (men)	41	121	0.4 (0.2-0.6)	0.5 (0.3-0.8)
Alcohol consumption				
Never	67	83	1.0	1.0
1-2 drinks/wk	37	56	0.8 (0.5-1.4)	0.8 (0.4-1.6)
3-6 drinks/wk	11	26	0.5 (0.2-1.1)	0.4 (0.1-1.1)
1-2 drinks/day	56	80	0.9 (0.5-1.4)	0.8 (0.5-1.5)
3 drinks/day or more	20	41	0.6 (0.3-1.1)	0.7 (0.3-1.6)
Family history of CTS				
None	160	260	1.0	1.0
Father/mother	15	22	1.1 (0.6-2.2)	1.3 (0.5-3.1)
Sibling	15	3	8.1 (2.3-29.2)	6.6 (1.5-29.4)
Rheumatoid arthritis				
No	160	267	1.0	1.0
Yes	30	19	2.6 (1.4-4.9)	2.2 (1.0-4.6)
Trigger finger				
No	157	271	1.0	1.0
Yes	33	15	3.8 (2.0-7.3)	2.7 (1.3-5.8)
Diabetes mellitus				
No	181	280	1.0	1.0
Yes	9	6	2.3 (0.8-6.7)	2.6 (0.7-8.7)
Renal failure				
No	187	282	1.0	
Yes	3	4	1.1 (0.2-5.1)	
Thyroid disorders				
No	163	253	1.0	
Yes	27	33	1.3 (0.7-2.2)	
Wrist fractures				
None	180	262	1.0	
At least one	11	24	0.7 (0.3-1.4)	
Smoking status				
Never	103	147	1.0	
Former	31	65	0.7 (0.4-1.1)	
Current	57	73	1.1 (0.7-1.7)	
Education level				
Below high school diploma	152	138	1.0	
High school diploma or higher	39	148	0.2 (0.2-0.4)	

*Multivariate unconditional logistic regression model adjusted for socio-occupational status, BMI, height, alcohol, family history of CTS, rheumatoid arthritis, trigger finger and diabetes mellitus (ie all the variables apart from education level that reached p < 0.1 at univariate analysis), as well as age and gender (the two variables used for frequency matching, which were included to reduce residual confounding) and center.

†The grouping of housewives with blue-collar workers was based on biomechanical considerations.

## Results

### Response

**Figure 1:**
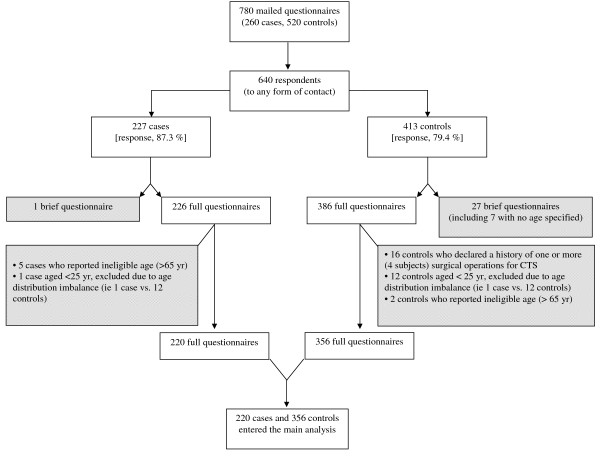
**Flow chart of data collection, exclusion criteria (excluded subjects are indicated in shaded boxes) and resulting groups used in data analysis**.

**Figure 2:**
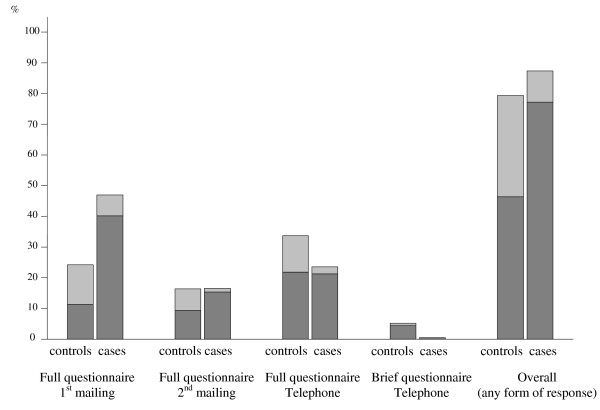
**Proportions of case/control respondents according to blue-collar (dark shading) and white-collar status (light shading)**.

Enrolment of the entire study population is summarized in Figures 1, 2 (see also Additional file 1). Of note, in only 1 'case' (and no 'control') was the questionnaire filled in by the next of kin. Response rates for cases and controls were not significantly different (p = 0.997, chi-square test) among the 13 centers. None of the centers showed less than 65% response for cases or controls. The 227 'cases' (and 413 'controls') who responded to the full or brief questionnaire represented 87% (and 79%) of all attempted contacts. After exclusion of responders to brief questionnaires and other non-eligible subjects (see Figure 1), 220 cases and 356 controls entered the main analysis. Mean ages were 48.3 ± 9.2 yr for women cases (n = 184) and 48.0 ± 9.1 yr for women controls (n = 286); 49.1 ± 8.8 yr for men cases (n = 36) and 51.9 ± 9.0 yr for men controls (n = 70). No difference in the distribution of cases and controls was found for age or gender, reflecting the frequency matching. Of note, the 45-54-yr age group accounted for 43% of all cases and 45% of controls. Cases and controls reported similar numbers of jobs (2.6 ± 1.5 for cases vs 2.5 ± 1.4 for controls among women; 3.0 ± 1.7 for cases vs 2.6 ± 1.3 for controls among men).

### Non-retired subjects (main analysis)

(see Additional file 2)

#### Socio-occupational and individual factors

The reference category comprised 153 white collar workers (19 cases and 134 controls), 85 of whom were administrative clerks (8 cases, 77 controls); no risk related to use of personal computers was apparent (stratifying by "no/occasional", "non-continuous" or "continuous" use; data not shown). Table 1 reports univariate and multivariate analysis of socio-occupational class and individual factors within the entire non-retired population. At unconditional logistic regression analysis, significant risk factors were blue-collar/housewife status (~7-fold vs white-collar), BMI ≥30 kg/m^2 ^(~3-fold), sibling history of CTS (~7-fold, with wide CI), and coexistence of trigger finger (~3-fold); rheumatoid arthritis was borderline (~2-fold). At gender stratification (Table 2), blue-collar status turned out to be a strong risk factor in both women (~9-fold) and men (~7-fold), while housewife status was also a risk factor (~4-fold) in women. Among women, it was feasible to perform age-stratification by tertiles (Table 3), which also happened to approximate pre-, peri- and post-menopausal age groupings. Blue-collar status appeared to be a risk factor in all three subgroups. Housewife status was an important factor in post-menopausal age. Individual factors appeared more relevant in younger women: in this subset, obesity was an important factor, whereas being relatively tall was associated with lower risk.

**Table 2:** Risk factors for surgically treated CTS among non-retired women and men

			**Univariate**	Multivariate*
	
**Women**	**Cases** **(n = 163)**	**Controls** **(n = 238)**	**OR (95% CI)**	**OR (95% CI)**
Socio-occupational status				
White collar	16	109	1.0	1.0
Blue collar	101	71	9.7 (4.9-19.2)	9.1 (4.8-17.4)
Housewife	46	58	5.4 (2.7-10.9)	4.4 (2.1-9.2)
BMI (kg/m^2^)				
<25	74	154	1.0	1.0
25-29	56	60	1.9 (1.2-3.1)	1.8 (1.1-3.1)
≥30	32	21	3.2 (1.7-6.0)	3.9 (1.9-8.1)
Height, cm				
<165 (women) or <175 (men)	126	144	1.0	1.0
≥165 (women) or ≥175 (men)	36	94	0.4 (0.3-0.7)	0.5 (0.3-0.8)
Parity				
None	29	54	1.0	1.0
1	42	80	1.0 (0.5-1.8)	0.8 (0.4-1.6)
2	62	76	1.5 (0.9-2.7)	1.4 (0.7-2.9)
3 or more	30	28	2.0 (1.0-4.0)	1.4 (0.6-3.3)

**Men**	**(n = 28)**	**(n = 48)**		

Socio-occupational status				
White collar	3	25	1.0	1.0
Blue collar	25	23	9.1 (2.1-39.2)	7.4 (1.7-33.2)
Housewife	--	--	--	--
BMI (kg/m^2^)				
<25	6	13	1.0	1.0
25-29	13	31	0.9 (0.3-2.9)	0.6 (0.1-2.4)
≥30	9	4	4.9 (0.9-25.8)	3.8 (0.6-25.7)
Height, cm				
<165 (women) or <175 (men)	23	21	1.0	1.0
≥165 (women) or ≥175 (men)	5	27	0.2 (0.0-0.6)	0.2 (0.0-0.6)
Parity				
None	--	--	--	--
1	--	--	--	--
2	--	--	--	--
3 or more	--	--	--	--

*Unconditional logistic regression models adjusted for socio-occupational status, BMI, height, age, center and (for women only) parity.

**Table 3:** Risk factors for surgically treated CTS in different age groups of non-retired women

			**Univariate**	Multivariate*
	
**Women <45 yr**	**Cases** **(n = 53)**	**Controls** **(n = 82)**	**OR (95% CI)**	**OR (95% CI)**
Socio-occupational status				
White collar	5	44	1.0	1.0
Blue collar	42	26	14.2 (4.2-48.6)	12.3 (3.9-39.3)
Housewife	6	12	4.4 (1.1-18.1)	2.5 (0.5-13.8)
BMI (kg/m^2^)				
<25	26	66	1.0	1.0
25-29	18	11	4.2 (1.7-10.5)	4.0 (1.3-12.5)
≥30	9	5	4.6 (1.3-15.7)	8.4 (1.6-43.9)
Height, cm				
<165	38	34	1.0	1.0
≥165	15	48	0.3 (0.1-0.6)	0.3 (0.1-0.7)

**Women 45-51 yr**	**(n = 51)**	**(n = 85)**		

Socio-occupational status				
White collar	8	43	1.0	1.0
Blue collar	33	22	8.1 (2.8-23.0)	7.7 (2.8-21.1)
Housewife	10	20	2.7 (0.9-8.1)	2.6 (0.8-8.6)
BMI (kg/m^2^)				
<25	25	54	1.0	1.0
25-29	16	25	1.4 (0.6-3.1)	1.6 (0.6-3.9)
≥30	10	4	5.4 (1.4-20.2)	6.0 (1.4-25.4)
Height, cm				
<165	41	56	1.0	1.0
≥165	10	29	0.5 (0.2-1.1)	0.6 (0.2-1.5)

**Women ≥52 yr**	**(n = 59)**	**(n = 71)**		

Socio-occupational status				
White collar	3	22	1.0	1.0
Blue collar	26	23	8.3 (1.9-35.7)	9.9 (2.3-42.6)
Housewife	30	26	8.5 (2.0-35.7)	9.5 (2.3-39.9)
BMI (kg/m^2^)				
<25	23	34	1.0	1.0
25-29	22	24	1.4 (0.6-3.0)	1.2 (0.5-2.9)
≥30	13	12	1.6 (0.6-4.2)	1.5 (0.5-4.4)
Height, cm				
<165	47	54	1.0	1.0
≥165	11	17	0.7 (0.3-1.8)	0.8 (0.3-2.2)

*Unconditional logistic regression models adjusted for socio-occupational status, BMI, height, age and center.

Table 4 reports results of an unconditional logistic regression model constructed for non-retired women to assess risks associated with blue-collar job titles (prevalent in the last 2 years or, for cases who changed task due to CTS, at the time of onset of symptoms). Raised OR were observed for food retail workers (~30-fold vs white-collar workers), waiters/bartenders (~20-fold), cooks (~17-fold), agricultural/horticultural workers (~13-fold), cleaners/domestic helpers (~9-fold), textile (mainly sewing-machine) workers (~9-fold), metal workers (~9-fold), packaging workers (~9-fold), and nursing/paramedical workers (~8-fold). Unsurprisingly given the relatively brief exposure period thought to be sufficient to precipitate onset of CTS symptoms [25], the magnitude of many of the professional associations turned out to be lower when job titles prevalent in the last 10 years or life-prevalent job titles were entered in the model (see Additional file 3).

**Table 4:** Risks of surgically treated CTS associated with job titles prevalent in the last 2 years* among non-retired women

				**Univariate**	**Multivariate**†
				
	**ISCO codes**	**Cases** **(n = 163)**	**Controls** **(n = 238)**	**OR (95% CI)**	**OR (95% CI)**
White collar	1, 21, 22 (not 2230), 23 (not 2332), 24, 311, 312, 315, 32 (not 3225 or 323), 341, 342, 343, 346, 41 (not 4131 or 4132), 42 (not 4211), 516	16	109‡	1.0	1.0
Food retail workers	5221	8	2	27.3 (4.3-173.2)	29.8 (5.6-159.7)
Miscellaneous blue-collar workers	4131, 4132, 71, 724, 73, 741, 7421, 7442, 822, 823, 8240, 825, 8266, 827, 8282, 8285, 931	14	5	19.1 (5.0-72.6)	21.4 (6.0-76.8)
Waiters/bartenders	5123	6	2	20.4 (3.2-129.0)	20.2 (3.4-119.5)
Cooks	5122	8	3	18.2 (3.7-88.7)	17.2 (3.7-78.8)
Agricultural/horticultural workers	61, 9211	10	4	17.0 (4.1-71.3)	13.3 (3.5-51.3)
Cleaners and domestic helpers	9131, 9132	13	8	11.1 (3.5-34.6)	9.2 (3.1-27.6)
Textile (mainly sewing-machine) workers	826	5	4	8.5 (1.9-37.7)	9.2 (2.0-42.4)
Metal workers	721, 722, 723, 812, 8211, 8281	5	4	8.5 (1.9-37.7)	9.0 (2.0-39.6)
Packaging workers	9320	7	5	9.5 (2.5-36.8)	8.7 (2.3-33.2)
Nurses and paramedical workers	2230, 3225, 323, 5132, 5133	9	7	8.8 (2.6-29.2)	7.9 (2.5-25.3)
Miscellaneous service sector workers	4211, 5141, 5220, 83, 911, 9133, 914, 915, 916	10	14	4.9 (1.8-13.4)	4.7 (1.7-12.9)
Housewives	5121	46	58	5.4 (2.7-10.9)	4.4 (2.1-9.1)
Tailors	743	4	6	4.5 (1.1-18.5)	3.5 (0.8-14.9)
Pre-primary school workers	2332, 3320, 5131	2	7	1.9 (0.4-10.3)	2.3 (0.4-12.5)

*For cases who changed task due to CTS, we considered the patient's job title at the time of onset of symptoms.

†Unconditional logistic regression model adjusted for the individual factors entered in the multivariate model reported in Table 2 (ie. BMI, height, parity, age and center).

‡Includes 1 student.

The PAR for blue-collar work was 77% (95%CI, 30%-99%) among men and 78% (95%CI, 63%-89%) among women. The PAR for housewives (calculated among women) was 56% (95%CI, 31%-77%).

#### Biomechanical risk factors

Ergonomists blind to case/control status deemed that reclassification of one or more self-reported risk factors was necessary for 264 participants: in particular, 250 participants (114 cases, 136 controls) had one or more factors reclassified as "no exposure"; 18 participants (9 cases, 9 controls) had at least one factor reclassified as an "exposure"; 4 of the participants had factors reclassified in both directions. The distribution of each of the six biomechanical factors among the socio-occupational groups and blue-collar job title categories is shown in Additional file 4. Table 5 reports the results of an unconditional logistic regression model in which blue-collar/housewife and white-collar status were crossed with presence of ≥1 biomechanical risk factor, a variable chosen as a broad marker of exposure. Raised risks were recorded for blue-collar/housewife status both with and without biomechanical risk factors. However, the point estimates of the OR were about 2-fold higher in the "exposed" subgroups (ie exposed white-collar workers and exposed blue-collar workers/housewives) in comparison with their "unexposed" counterparts. Table 6 reports univariate and multivariate analysis of risks associated with different numbers/combinations of the factors (based on ergonomic and frequency considerations). No dose-response relation could be observed for exposure to increasing numbers of factors. However, incremental OR point estimates were apparent for combinations of biomechanical factors that included frequent repetitivity and sustained force.

**Table 5:** Risk of surgically treated CTS in socio-occupational categories stratified according to biomechanical exposure (in terms of at least 1 plausible risk factor)

			**Univariate**	Multivariate*
			
	**Cases**	**Controls**	**OR (95% CI)**	**OR (95% CI)**
White-collar workers				
Not exposed	16	122	1.0	1.0
Exposed	3	12	1.9 (0.5-7.6)	2.1 (0.5-9.5)
Blue-collar/housewife status				
Not exposed	66	93	5.4 (2.8-10.3)	5.1 (2.6-9.9)
Exposed	106	59	13.7 (6.6-28.3)	12.8 (6.4-25.3)

*Unconditional logistic regression model adjusted for the individual factors entered in the multivariate model reported in Table 1 (ie BMI, height, alcohol, family history of CTS, rheumatoid arthritis, trigger finger and diabetes), as well as age, gender and center.

### Retired subjects

The reference category comprised 26 ex-white collar workers (4 cases, 22 controls). At multivariate analysis (adjusting for the individual factors considered in the model reported in Table 1), history of blue-collar work reached borderline significance as a predictor of surgically treated CTS (OR, 4.2; 95% CI, 1.0-17.5). Number considerations precluded further analysis.

## Discussion

This multicenter case-control study can be considered population-based since the cases were randomly drawn from comprehensive (obligatorily compiled) records of all operations performed by any Italian public or private hospital for patients residing within the territory covered by each administrative body. The work has the distinguishing feature that it considered both non-occupational and occupational factors, including job titles and biomechanical risk factors. Taken together, the results indicate that within the general population under study in our Italian centers risk of requiring surgically treated CTS resides mainly in the broad socio-occupational class of blue-collar workers, where manual work predominates. Notably, high OR were recorded for manual jobs in both the non-industrial and industrial sectors.

After adjusting for BMI, height, and other non-occupational variables, the broad socio-occupational class of blue-collar workers and housewives appeared to have a 7-fold (point estimate) risk of surgical treatment for CTS, as compared with white-collar workers. Blue-collar work was associated with raised risks in both women and men. These findings are broadly in line with the results of a study by Rossignol *et al *of surgical interventions for CTS recorded on the Quebec Health Insurance database [30]. In the present study, full-time housewives (an important socio-occupational category accounting for 29% of all adult women in Italy) [31] also had a raised risk (4-fold point estimate). Unsurprisingly, biomechanical exposure was common among both blue-collar workers and housewives (see Additional file 4). After adjusting for potential confounders, blue-collar worker/housewives who reported at least one plausible biomechanical risk factor appeared to have about twice the risk of surgically treated CTS with respect to their "unexposed" counterparts. Furthermore, the few biomechanically "exposed" white-collar workers showed a non-significant point estimate of ~2-fold risk with respect to "unexposed" white-collar workers (Table 5). Taken together, these observations seem to spotlight the etiologic relevance of exposure to ergonomic factors within an occupational context. Indeed, substantial PAR were recorded for women/men blue-collar workers and housewives, broadly in line with those (adjusted only for age and sex) recorded for manual workers in Montreal [30].

It should be underlined that due to the collection of only binary biomechanical data, it cannot be assumed that those workers/housewives who did not report risk factors were truly unexposed. Thus, it is difficult to distinguish a possible biomechanical contribution in the apparently "unexposed" subjects from other socio-occupationally related (eg psychosocial) factors. Of note, malingering is unlikely to have exerted much influence on these findings given the case definition of surgically treated CTS: any "invented" cases would have to pass through a stringent preoperative clinical workup including nerve conduction studies.

Few non-cross-sectional analytical studies have assessed associations between job titles and risk of CTS in the general population. In the present work, analysis of job titles was feasible only for women (due to number considerations). The findings must be interpreted in the context of the employment characteristics of the general population in the various centers. In particular, our reference category of women white-collar workers mainly comprised clerks who appeared to have little biomechanical exposure. It should be underlined that this study does not provide information regarding the highly relevant occupational category of data processors [30], who were poorly represented in the general population of the areas under study. Moreover, many industrial job titles were poorly represented due to regional employment characteristics (the 95% CI for specific job titles and other occupational subgroups tended to be wide due to limited absolute numbers). Although most studies of occupational risk of CTS have focused on industrial settings [17], some at-risk "non-industrial" occupational job titles have also been reported, including housekeepers/cleaners, food/beverage service workers, grocery store workers, postal workers, health workers, lorry/bus drivers, and child care workers [14,30,32]. In the present work, raised risks of surgically treated CTS were recorded in several clearly non-industrial blue-collar categories: food retail workers, waiters/bartenders, cooks, agricultural/horticultural workers (including many in the fruit-growing sector), cleaners/domestic helpers, and nursing/paramedical workers (with point estimates between about 8 and 30, albeit with wide 95% CI). In a seminal article based on a hospital case series, Phalen noted that the majority of his patients were housekeepers or cooks [33]. More recently, Rossignol *et al *found that housekeeping occupations (including both commercial and domestic categories) appeared to be associated with a particularly risk of surgically treated CTS [30]. A previous Italian case-control study focusing on job tasks reported significantly raised risks of hospital-treated CTS for waiters/bartenders and cooks [34]. Of note, bartenders in Italy are commonly exposed to repetitive wrist actions (a characteristic jerk) to operate espresso coffee machines, and this factor could at least partially explain the recorded excess risk. From an ergonomic standpoint, it is noteworthy that many plausible biomechanical risk factors were reported by cooks, waiters/bartenders, food retail workers and agricultural/horticultural workers, but not by nurses/paramedics or cleaners/domestic helpers (see Additional file 4). However, it is possible that the collection of binary variables for biomechanical risk factors missed frequent medium/low-grade exposures. In general, we think that non-industrial job titles, particularly in the service sector) deserve more attention in CTS risk evaluation studies, including ergonomic evaluations.

The high OR recorded for nurses and paramedical workers could be attributable to several factors in addition to biomechanical risk factors, including possible facilitated access to surgical treatment for hospital workers. Nurse anesthetists are thought to be especially exposed to risk factors for CTS [35], but we have no way of knowing how many, if any, of the cases in the present study had this specific job title.

The roughly 4-fold increases of risk recorded for full-time housewives may also be at least partially attributed to ergonomic factors (despite the relative paucity of reported biomechanical risk factors). Although some residual confounding with parity and especially BMI is possible, we think that the contribution of domestic cleaning activities deserves consideration. In particular, it is reasonable to suppose that chronic biomechanical exposure might at least partially explain the high risk recorded among older housewives (Table 3). A case-control study on clinically/electromyographically diagnosed CTS among Chinese women in Beijing concluded that certain manual household tasks could be associated with increased risk of CTS [13].

Although the exploratory analysis of plausible self-reported biomechanical risk factors (Table 6) was limited by the binary data collection and frequency considerations, the results are intriguing. The lack of a dose-response relation for exposure to different numbers of risk factors is in line with the concept that some of these factors selected from the available ergonomic literature [14-17] are particularly relevant [17,29]. Number considerations permitted us to explore combinations of just four of the main candidates: sustained force, frequent repetitivity, awkward posture, and palmar compression (unfortunately, use of vibratory tools was relatively rare in the study population). We dedicated particular attention to force and frequency because these two aspects form the basis of the official instrument developed by the American Conference of Governmental Industrial Hygienists (ACGIH) for objective evaluation of biomechanical hand/wrist load (and values exceeding the hand activity limit have been associated with high rates of symptomatic CTS [36]). Strikingly, combinations that included these two factors (with or without awkward posture and palmar compression) were always associated with particularly high OR (Table 6). These findings reinforce concepts that have previously emerged from cross-sectional and cohort studies regarding the etiologic relevance of forceful and/or very repetitive manual work [14-16,36,37].

**Table 6:** Risks of surgically treated CTS associated with different numbers/combinations of biomechanical risk factors (use of vibratory tools; forceful hand/wrist movements; frequent repetitive hand/wrist movements; uncomfortable hand postures; frequent pinching actions; skin compression) among non-retired subjects

			**Univariate**	Multivariate*
			
	**Cases** **(n = 191)**	**Controls** **(n = 286)**	**OR ** **(95% CI)**	**OR** **(95% CI)**
No risk factor†	82	215	1.0	1.0
1 risk factor	48	42	3.0 (1.8-4.9)	2.8 (1.6-4.8)
*Either force or frequency*	*41*	*33*	*3.3 (1.9*-*5.6)*	*3.2 (1.7*-*5.8)*
*Any other factor*	*7*	*9*	*2.0 (0.7*-*5.7)*	*1.4 (0.5*-*4.6)*
2 risk factors	23	9	6.7 (2.9-15.6)	7.1 (2.9-17.4)
*Any 2 factors from: force, frequency and posture*	*15*	*4*	*9.8 (3.0*-*31.9)*	*10.3 (3.0-35.1)*
*Other combinations (of 2 risk factors)*	*8*	*5*	*4.2 (1.3*-*13.4)*	*4.0 (1.1*-*14.8)*
3-4 risk factors	31	17	4.8 (2.4-9.3)	4.5 (2.1-9.3)
*At least 3 factors from: force, frequency, posture and compression*	*16*	*3*	*14.0 (3.7*-*52.2)*	*14.9 (3.9-57.6)*
*Other combinations (of 3 or 4 risk factors)*	*15*	*14*	*2.8 (1.3*-*6.1)*	*2.2 (0.9*-*5.5)*
5-6 risk factors	7	3	6.1 (1.5-24.8)	5.0 (1.1-22.6)

*The results derive from two separate multivariate unconditional logistic regression models (evaluating numbers/combinations of risk factors). Each model was adjusted for the individual factors entered in the multivariate model reported in Table 1 (ie BMI, height, alcohol, family history of CTS, rheumatoid arthritis, trigger finger and diabetes mellitus), as well as age, gender and center.

†Reference category.

Regarding non-occupational variables, obesity has already been widely implicated as a risk factor for CTS in both cohort and case-control studies [3,5] with global increases in risk broadly similar to those recorded in the present study. Among women, obesity-related risk of surgically treated CTS appeared to be highest in the lowest age tertile (<45 years) (Table 3). A study exploring the relationship between obesity, age and CTS among patients registered at a neurophysiology department [38] found higher obesity-related risks in younger age groups for both women and men (the limited number of men in our study precluded meaningful analysis). Such age-related differences could be attributed to different underlying pathogenetic mechanisms for CTS in younger and older people [38]. By contrast, at least among the women, blue-collar socio-occupational status appeared to be a risk factor at all ages.

In two hospital-based case-control studies, taller individuals appeared to have a lower risk of CTS [10,11]. In the present population-based study, relatively tall women and men (as defined by the upper tertiles of women and men controls) also turned out to be associated with lower risk (with point estimates of about 0.5 for women over 165 cm and men over 175 cm tall), even after adjusting for BMI and other possible risk factors. Even inclusion of level of education in the model (data not shown) to try to reduce social class-related bias [39] did not substantially change the results. Remarkably, even among white-collar workers, height appeared to be associated with reduced risk (point estimate, ~0.2; data not shown). No interaction was found between height and BMI (data not shown). Height could be a marker of specific anthropometric characteristics of the forearm, wrist and hand [6]. Square wrist shape is a proposed risk factor for CTS [40]. Height-related size variations of tendons and muscles might also play a role.

Regarding parity, a roughly 2-fold (point estimate) excess risk of surgically treated CTS was recorded at univariate analysis for women with three or more children, but no significant association was detectable at multivariate analysis. An excess of risk was reported in a nested case-control study [41]. Our results should not have been affected by CTS during pregnancy (a phenomenon presumably influenced by physiological changes in hormone production and body mass): in line with the findings of a dedicated cohort study [42], very few cases in the study population (n = 4; not shown) were submitted to surgery during pregnancy or in the year after childbirth. Along with possible residual confounding with BMI, biomechanical exposure during domestic work could at least partially explain the apparent excess risk associated with high parity (of note, women workers who had more children reported more hours of domestic chores; see Additional file 2). Alternative explanations could involve long-term pathophysiologic effects of multiple pregnancies.

Lack of association between smoking habits and CTS was expected [9]. Our results provide some indication that light consumption of alcohol (3-6 drinks per week) might conceivably be protective: we think that information on drinking habits should be collected in epidemiologic studies of CTS, especially in view of the anti-inflammatory properties of red wine [43], which is widely consumed in our geographic setting. Another possible association which needs to be tested in larger studies regards surgical treatment for CTS in siblings. This putative risk factor could be attributed either to genetic and/or familial environmental factors. Unfortunately, our questionnaires did not provide information on the number of subjects with or without siblings (or numbers of siblings). Nevertheless, the finding of an almost 7-fold excess risk in subjects with an affected sibling is particularly interesting in the light of a study of female twins which suggested that up to half the risk of CTS in women may be genetically determined [7].

Remarkably similar OR values were generally recorded at univariate and multivariate analysis suggesting a relative lack of confounding among different risk factors (except perhaps for high BMI in young women). In all age groups, both men and women blue-collar workers (in industrial/non-industrial settings) turned out to be at increased risk of surgical treatment for CTS. We think it likely that biomechanical factors (which are more frequently encountered in manual blue-collar occupations) influence onset of CTS in both genders at almost any age.

### Study limitations

It should be underlined that the sample size was not large enough to address many of the study objectives with confidence. The suggestive findings emerging from the present work require confirmation in appropriately sized studies, and also in geographic settings with different employment characteristics. Indeed, as noted above, many relevant job titles were either poorly represented or absent in our limited sample. For instance, the very high risks recorded for the broad blue-collar category may partially be attributed to the relative absence of intensive keyboard/mouse users such as data processors in the study sample.

In view of the relatively good but not ideal response (74%) to the full questionnaire among potential controls, non-response bias may have influenced the main findings regarding socio-occupational status. Although this factor could have led to a slight overestimate of the risks associated with blue-collar/housewife status, it seems unlikely that the 7-fold excess risk could be mainly attributable to blue-collar/housewife non-responders among controls. Recall bias also requires consideration in case-control studies, especially when participants are aware of objectives and their case/control status. Although differential recall may not have influenced reporting of socio-occupational category or job titles, it could have affected the analyses involving biomechanical risk factors. Interpretation of these analyses is also affected by the decision to use binary variables for biomechanical data collection and classification (see discussion above). Expert ergonomists blindly reviewed the plausibility of the self-reported biomechanical risk factors and frequently modified exposure status; it should be borne in mind that the study participants received no guidance on how to interpret the descriptions of the biomechanical factors in the questionnaire (eg regarding what constitutes "frequent pinching actions"). Remarkably, a supplemental analysis (data not shown) of the self-reported biomechanical risk factors before the ergonomists' plausibility evaluations generated results substantially similar to those reported in Table 6.

The risk estimates reported in the present study regard surgically-treated CTS and cannot automatically be extended to all clinically relevant cases of CTS. In the general population of Siena (Tuscany) [22,44], surgical treatment seems to be performed in no more than about half the patients with an electromyographically confirmed diagnosis of symptomatic CTS (Table 7). In Siena at least, surgically treated patients [45] seem to have a more severe clinical and electrophysiological profile than untreated patients. In other respects, however, the characteristics of the two groups [45] appear broadly similar apart from a slightly lower level of education among the patients not submitted to surgical treatment. This knowledge seems to attenuate the legitimate concern that blue-collar workers, housewives and mothers of several children might have greater incentives to undergo surgical decompression of the median nerve in order to remain fit for essential manual activities (whereas male white-collar workers might be better placed to avoid particular tasks and postpone or avoid surgical treatment). We think that use of 'surgically-treated CTS' as a case definition may provide (in Italy at least) a heuristic tool to help spotlight more clinically severe and socially relevant disease. Nevertheless, it must be stressed that a case definition of 'surgically-treated CTS' will presumably lead to identification of risk factors for having "CTS-related troubles" severe enough to warrant surgery (rather than just experiencing the disease itself). The higher risks recorded for manual workers could at least partially stem from this consideration.

**Table 7:** Comparison of crude and sex-specific incidence rates (per 100,000 person-years) of CTS diagnosed by clinical symptoms plus electromyography (EMG) in the province of Siena for 1997-1998 [44] with in-hospital rates recorded [22] in the same province and period

		**Symptoms/EMG **[44]	**In-hospital **[22]	**Ratio**
1997	Women	478.6	244.2	2.0
	Men	161.4	68.4	2.4
	Overall	326.5	159.9	2.0

1998	Women	497.8	193.9	2.6
	Men	178.5	57.7	3.1
	Overall	344.5	128.5	2.7

## Conclusion

In summary, this population-based case-control study highlights the relevance of biomechanical exposures encountered in non-industrial as well as industrial blue-collar work as risk factors for surgically treated CTS in both sexes, and among different age groups of women. By contrast, associations with factors such as BMI and height seem to vary with age and gender. Until now, attention has mainly been focused on manual work in the industrial sector. We think more attention should be dedicated to evaluation of possible causal contributions of certain manual job tasks in the non-industrial sector in the etiology of severe CTS. If confirmed in larger studies, such considerations could be pertinent for prevention of socially/clinically relevant morbidity, and also for adjudication of workers' compensation insurance claims.

## Competing interests

The authors declare that they have no competing interests.

## Authors' contributions

SM, AB and GM designed the study with SC, MdO, FG, RL, and MBo.

AM, TM, CS, SB, MBr, RG, PGB, MPC, AMC, FF, SF, MdO, PG, PFM were responsible for supervising data collection in the different centers. SM, SC and GC were responsible for data analysis. SM, SC and RMTC drafted the manuscript and contributed to interpretation, together with AB, MF, FZ, MBo and FSV. FSV supervised the entire work. All authors critically revised the manuscript. All authors read and approved the final manuscript.

## Pre-publication history

The pre-publication history for this paper can be accessed here:



## Supplementary Material

Additional file 1 **Distribution of response modalities among the 780 attempted contacts (260 for cases, 520 for controls), and distributions of level of education and professional category among respondents**. Click here for file

Additional file 2 **Summary statistics of selected individual factors (and hours of housework) according to gender and socio-occupational category**. Click here for file

Additional file 3 **Risks of surgically treated CTS associated with job titles prevalent in the last 10 years and lifetime among non-retired women**. Click here for file

Additional file 4 **Numbers (percentages) of workers exposed to different biomechanical risk factors by socio-occupational status and blue-collar job titles**. Click here for file
