# Supplementary material for: Risk factors for operated carpal tunnel syndrome: a multicenter population-based case-control study
Source: BMC Public Health. 2009 Sep 16;9:343. doi: 10.1186/1471-2458-9-343 (PMC2761403; doi:10.1186/1471-2458-9-343)
Supplement: Additional file 1 — Distribution of response modalities among the 780 attempted contacts (260 for cases, 520 for controls), and distributions of level of education and professional category among respondents. [file 1471-2458-9-343-S1.pdf]

**Additional file 1.** Distribution of response modalities among the 780 attempted contacts (260 for cases, 520 for controls), and distributions of level of education and professional category among respondents

| | 'Cases' | | | 'Controls' | | |
|---|---|---|---|---|---|---|
| | **Overall responses n/N (%)** | No school diploma n/N (%) | Blue-collar workers n/N (%) | **Overall responses n/N (%)** | No school diploma n/N (%) | Blue-collar workers n/N (%) |
| **Full questionnaire** | **226/260 (86.9)** | 175/226 (77.4) | 195/221* (88.2) | **386/520 (74.2)** | 203/386 (52.6) | 219/383* (57.2) |
| ***1st mailing*** | ***122/260 (46.9)*** | *86/122 (70.5)* | *100/117* (85.5)* | ***126/520 (24.2)*** | *49/126 (38.9)* | *59/126 (46.8)* |
| ***2nd mailing*** | ***43/260 (16.5)*** | *36/43 (83.7)* | *40/43 (93.0)* | ***85/520 (16.3)*** | *47/85 (55.3)* | *47/82* (57.3)* |
| ***Telephone*** | ***61/260 (23.5)*** | *53/61 (86.9)* | *55/61 (90.2)* | ***175/520 (33.7)*** | *107/175 (61.1)* | *113/175 (64.6)* |
| **Brief questionnaire** | **1/260 (0.4)** | 1/1 (100.0) | 1/1 (100.0) | **27/520 (5.2)** | 20/21* (95.2) | 18/22* (81.8) |
| **Any form of response** | **227/260 (87.3)** | 176/227 (77.5) | 196/222* (88.3) | **413/520 (79.4)** | 223/407* (54.8) | 237/405* (58.5) |

*These denominators differ from the overall numbers of respondents due to incomplete information in the replies.
