# Supplementary material for: Risk factors for operated carpal tunnel syndrome: a multicenter population-based case-control study
Source: BMC Public Health. 2009 Sep 16;9:343. doi: 10.1186/1471-2458-9-343 (PMC2761403; doi:10.1186/1471-2458-9-343)
Supplement: Additional file 2 — Summary statistics of selected individual factors (and hours of housework) according to gender and socio-occupational category. [file 1471-2458-9-343-S2.pdf]

Additional file 2. Summary statistics of selected individual factors (and hours of housework) according to gender and socio-occupational category

	Women									Men					
	White Collar			Blue Collar			Housewives			White Collar			Blue Collar		
	**Cases** N=16	**Controls** N=109	**Overall** N=125	**Cases** N=101	**Controls** N=71	**Overall** N=172	**Cases** N=46	**Controls** N=58	**Overall** N=104	**Cases** N=3	**Controls** N=25	**Overall** N=28	**Cases** N=25	**Controls** N=23	**Overall** N=48
	**mean±sd**	**mean±sd**	**mean±sd**	**mean±sd**	**mean±sd**	**mean±sd**	**mean±sd**	**mean±sd**	**mean±sd**	**mean±sd**	**mean±sd**	**mean±sd**	**mean±sd**	**mean±sd**	**mean±sd**
BMI, kg/m^2	26.2±5.9	23.0±3.6	23.5±4.1	25.9±4.5	23.7±3.4	25.0±4.2	26.8±5.9	26.5±5.7	26.6±5.7	25.7±3.9	26.8±3.0	26.7±3.1	28.4±3.7	26.3±2.8	27.4±3.4
Height, cm	161.9±5.2	163.4±6.3	163.2±6.2	159.2±6.1	162.4±5.5	160.5±6.0	160.7±5.3	160.8±5.6	160.7±5.5	170.7±2.3	175.1±5.7	174.6±5.6	171.5±6.2	173.7±6.7	172.5±6.5
Parity, n	1.4±1.1	1.1±0.9	1.1±1.0	1.4±1.1	1.5±1.1	1.5±1.1	2.3±1.4	1.8±1.2	2.0±1.3	—	—	—	—	—	—
Housework, h/day	3.9±2.7	3.7±2.7	3.8±2.7	5.5±4.4	4.4±2.9	5.0±3.9	8.9±4.3	7.8±3.5	8.3±3.9	0.7±0.6	0.9±1.4	0.9±1.3	0.7±1.1	0.3±0.8	0.5±1.0
	**n (%)**	**n (%)**	**n (%)**	**n (%)**	**n (%)**	**n (%)**	**n (%)**	**n (%)**	**n (%)**	**n (%)**	**n (%)**	**n (%)**	**n (%)**	**n (%)**	**n (%)**
High educational status*	8 (50.0)	87 (79.8)	95 (76.0)	15 (14.9)	21 (29.6)	36 (20.9)	7 (15.2)	15 (25.9)	22 (21.2)	3 (100.0)	20 (80.0)	23 (82.1)	6 (24.0)	5 (21.7)	11 (22.9)
Drinkers	10 (62.5)	74 (67.9)	84 (67.2)	63 (62.4)	49 (69.0)	112 (65.1)	25 (54.4)	35 (60.3)	60 (57.7)	3 (100.0)	24 (96.0)	27 (96.4)	23 (92.0)	21 (91.3)	44 (91.7)
Current smokers	1 (6.3)	27 (24.8)	28 (22.4)	40 (39.6)	21 (29.6)	61 (35.5)	6 (13.0)	13 (22.4)	19 (18.3)	1 (33.3)	7 (29.2)	8 (29.6)	9 (36.0)	5 (21.7)	14 (29.2)
Family history of CTS															
Father/mother	0 (0.0)	12 (11.0)	12 (9.6)	11 (11.1)	5 (7.0)	16 (9.4)	2 (4.4)	0 (0.0)	2 (1.9)	0 (0.0)	3 (12.0)	3 (10.7)	2 (8.0)	2 (8.7)	4 (8.3)
Sibling	0 (0.0)	2 (1.8)	2 (1.6)	7 (7.0)	0 (0.0)	7 (4.1)	6 (13.0)	1 (1.8)	7 (6.8)	—	—	—	2 (8.0)	0 (0.0)	2 (4.2)
Most frequent comorbidities															
Rheumatoid arthritis	0 (0.0)	7 (6.4)	7 (5.6)	15 (15.0)	4 (5.6)	19 (11.1)	10 (21.7)	3 (5.2)	13 (12.5)	1 (33.3)	0 (0.0)	1 (3.6)	4 (16.0)	5 (21.7)	9 (18.8)
Trigger finger	0 (0.0)	1 (0.9)	1 (0.8)	22 (22.0)	7 (9.9)	29 (17.0)	8 (17.4)	5 (8.6)	13 (12.5)	1 (33.3)	2 (8.0)	3 (10.7)	2 (8.0)	0 (0.0)	2 (4.2)
Thyroid disorders	0 (0.0)	15 (13.8)	15 (12.0)	15 (15.0)	7 (9.9)	22 (12.9)	10 (21.7)	10 (17.2)	20 (19.2)	0 (0.0)	1 (4.0)	1 (3.6)	2 (8.0)	0 (0.0)	2 (4.2)
At least 1 wrist fracture	0 (0.0)	7 (6.4)	7 (5.6)	4 (4.0)	6 (8.5)	10 (5.8)	2 (4.4)	4 (6.9)	6 (5.8)	1 (33.3)	6 (24.0)	7 (25.0)	4 (16.0)	1 (4.4)	5 (10.4)

*High school diploma or higher.
