# Supplementary material for: Risk factors for operated carpal tunnel syndrome: a multicenter population-based case-control study
Source: BMC Public Health. 2009 Sep 16;9:343. doi: 10.1186/1471-2458-9-343 (PMC2761403; doi:10.1186/1471-2458-9-343)
Supplement: Additional file 3 — Risks of surgically treated CTS associated with job titles prevalent in the last 10 years and lifetime among non-retired women. [file 1471-2458-9-343-S3.pdf]

Additional file 3. Risks of surgically treated CTS associated with job titles prevalent in the last 10 years and lifetime among non-retired women

	Last 10 years				Lifetime			
			Univariate	Multivariate*			Univariate	Multivariate*
	Cases (n = 169)	Controls (n = 249)	OR (95% CI)	OR (95% CI)	Cases (n = 184)	Controls (n = 285)	OR (95% CI)	OR (95% CI)
White collar	20	108†	1.0	1.0	22	120	1.0	1.0
Food retail workers	8	2	21.6 (3.6–129.2)	21.9 (4.1–116.9)	9	2	24.5 (4.2–144.5)	24.1 (4.7–123.3)
Miscellaneous blue-collar workers	13	6‡	11.7 (3.5–38.7)	11.4 (3.4–38.0)	14	6	12.7 (3.9–41.3)	12.1 (4.0–37.0)
Waiters/bartenders	6	4	8.1 (2.0–33.5)	7.6 (1.8–31.2)	4	7	3.1 (0.8–11.8)	3.5 (0.9–13.8)
Cooks	6	4	8.1 (2.0–33.5)	8.7 (2.0–37.8)	4	5	4.4 (1.1–18.0)	3.8 (0.9–16.3)
Agricultural/horticultural workers	10	6	9.0 (2.7–30.0)	7.0 (2.1–23.0)	13	6	11.8 (3.6–38.4)	9.2 (3.0–28.4)
Cleaners and domestic helpers	12	13	5.0 (1.9–13.1)	4.1 (1.5–11.1)	9	9	5.5 (1.9–16.0)	4.1 (1.4–12.1)
Textile (mainly sewing-machine) workers	4	3	7.2 (1.4–36.6)	9.9 (1.8–55.2)	7	11	3.5 (1.2–10.2)	3.3 (1.1–10.0)
Metal workers	5	3	9.0 (1.9–43.7)	9.8 (2.0–47.5)	3	8	2.0 (0.5–8.4)	2.0 (0.5–8.8)
Packaging workers	6	5	6.5 (1.7–24.6)	5.7 (1.5–21.6)	5	7	3.9 (1.1–13.7)	4.9 (1.3–18.7)
Nurses and paramedical workers	6	11	2.9 (1.0–9.0)	2.3 (0.8–7.2)	8	16	2.7 (1.0–7.3)	2.7 (1.0–7.3)
Miscellaneous service sector workers	15	17	4.8 (2.0–11.6)	4.7 (1.9–11.5)	26	18	7.9 (3.4–18.1)	7.0 (3.2–15.2)
Housewives	50	56	4.8 (2.5–9.3)	3.9 (1.9–7.7)	50	51	5.3 (2.8–10.2)	4.7 (2.4–9.3)
Tailors	6	5	6.5 (1.7–24.6)	6.2 (1.6–23.9)	9	9	5.5 (1.9–16.0)	6.2 (2.1–18.7)
Pre-primary school workers	2	6	1.8 (0.3–9.6)	1.9 (0.3–10.9)	1	10	0.5 (0.1–4.5)	0.7 (0.1–5.5)

NOTE: Discrepancies in the numbers of cases and controls in these two analyses depend on the different definitions of prevalent job titles (more subjects satisfy a definition based on lifetime prevalence than one based on “prevalent in the last 10 years” or (as in the main analysis) “prevalent in the last 2 years”).

*Unconditional logistic regression models adjusted for the individual factors entered in the multivariate model reported in Table 2 (ie. BMI, height, parity, age and center).

†Includes 2 students.

‡Includes 1 unemployed blue collar worker.
