# Supplementary material for: Risk factors for operated carpal tunnel syndrome: a multicenter population-based case-control study
Source: BMC Public Health. 2009 Sep 16;9:343. doi: 10.1186/1471-2458-9-343 (PMC2761403; doi:10.1186/1471-2458-9-343)
Supplement: Additional file 4 — Numbers (percentages) of workers exposed to different biomechanical risk factors by socio-occupational status and blue-collar job titles. [file 1471-2458-9-343-S4.pdf]

Additional file 4. Numbers (percentages) of workers exposed to different biomechanical risk factors by socio-occupational status and blue-collar job titles

	**White collar (N=153)**	**Housewives (N=104)**	**Blue-collar (N=220)**	*Food retail workers (N=10)*	*Agricultural/horticultural workers (N=18)*	*Waiters/bartenders (N=9)*	*Miscellaneous blue-collar workers (N=36)*	*Cooks (N=11)*	*Nurses and paramedical workers (N=17)*	*Cleaners and domestic helpers (N=21)*	*Textile (mainly sewing-machine) workers (N=9)*	*Miscellaneous service sector workers (N=26)*	*Packaging workers (N=13)*	*Drivers (N=8)*	*Metal workers (N=23)*	*Tailors (N=10)*	*Pre-primary school workers (N=9)*
**Not exposed**	**138**	**55**	**104**	*1*	*6*	*1*	*17*	*0*	*16*	*16*	*1*	*18*	*4*	*8*	*2*	*5*	*9*
	**(90%)**	**(53%)**	**(47%)**	*(10%)*	*(33%)*	*(11%)*	*(47%)*	*(0%)*	*(94%)*	*(76%)*	*(11%)*	*(69%)*	*(31%)*	*(100%)*	*(9%)*	*(50%)*	*(100%)*
**Vibratory tools**	**1**	**0**	**24**	*0*	*2*	*0*	*7*	*0*	*1*	*0*	*1*	*0*	*0*	*0*	*13*	*0*	*0*
	**(0.7%)**	**(0%)**	**(11%)**	*(0%)*	*(11%)*	*(0%)*	*(19%)*	*(0%)*	*(6%)*	*(0%)*	*(11%)*	*(0%)*	*(0%)*	*(0%)*	*(57%)*	*(0%)*	*(0%)*
**Forceful hand/wrist**	**1**	**47**	**69**	*6*	*10*	*3*	*14*	*11*	*1*	*3*	*2*	*2*	*1*	*0*	*14*	*2*	*0*
**movements**	**(0.7%)**	**(45%)**	**(31%)**	*(60%)*	*(56%)*	*(33%)*	*(39%)*	*(100%)*	*(6%)*	*(14%)*	*(22%)*	*(8%)*	*(8%)*	*(0%)*	*(61%)*	*(20%)*	*(0%)*
**Frequent repetitive**	**10**	**0**	**65**	*8*	*4*	*5*	*6*	*11*	*1*	*2*	*7*	*3*	*7*	*0*	*8*	*3*	*0*
**hand/wrist movements**	**(7%)**	**(0%)**	**(30%)**	*(80%)*	*(22%)*	*(56%)*	*(17%)*	*(100%)*	*(6%)*	*(10%)*	*(78%)*	*(12%)*	*(54%)*	*(0%)*	*(35%)*	*(30%)*	*(0%)*
**Uncomfortable hand**	**4**	**3**	**63**	*4*	*1*	*8*	*10*	*11*	*1*	*2*	*5*	*4*	*4*	*0*	*9*	*4*	*0*
**postures**	**(3%)**	**(3%)**	**(29%)**	*(40%)*	*(6%)*	*(89%)*	*(28%)*	*(100%)*	*(6%)*	*(10%)*	*(56%)*	*(15%)*	*(31%)*	*(0%)*	*(39%)*	*(40%)*	*(0%)*
**Frequent pinching**	**1**	**1**	**43**	*2*	*3*	*0*	*6*	*1*	*1*	*1*	*6*	*4*	*6*	*0*	*10*	*3*	*0*
	**(0.7%)**	**(1%)**	**(20%)**	*(20%)*	*(17%)*	*(0%)*	*(17%)*	*(9%)*	*(6%)*	*(5%)*	*(67%)*	*(15%)*	*(46%)*	*(0%)*	*(43%)*	*(30%)*	*(0%)*
**Skin compression**	**0**	**1**	**42**	*4*	*6*	*3*	*7*	*7*	*1*	*2*	*1*	*2*	*2*	*0*	*6*	*1*	*0*
	**(0%)**	**(1%)**	**(19%)**	*(40%)*	*(33%)*	*(33%)*	*(19%)*	*(64%)*	*(6%)*	*(10%)*	*(11%)*	*(8%)*	*(15%)*	*(0%)*	*(26%)*	*(10%)*	*(0%)*

Note: Individual workers can have more than 1 exposure.
